# How to Set Working Cannula in Endoscopic‐Assisted Transforaminal Lumbar Interbody Fusion: A Morphometric Analysis Based on Computed Tomography

**DOI:** 10.1111/os.14239

**Published:** 2024-09-10

**Authors:** Conghui Zhou, Junsheng Lou, Yunpeng Fan, Ziyi Guo, Honghao Shen, Mengran Jin, Junsong Wu

**Affiliations:** ^1^ Department of Orthopedic Surgery The First Affiliated Hospital, Zhejiang University School of Medicine Hangzhou China; ^2^ Shenzhen Pingle Orthopedics Hospital Shenzhen China

**Keywords:** Computed Tomography, Endoscopic‐Assisted Transforaminal Lumbar Interbody Fusion (Endo‐TLIF), Exiting Nerve Root, Intervertebral Foramen, Three‐Dimensional Reconstruction

## Abstract

**Objectives:**

There is a high risk of nerve root injury during endoscopic‐assisted transforaminal lumbar interbody fusion (Endo‐TLIF). This study used computed tomography (CT) imaging to assess the relationship between the exiting nerve root and its surroundings, and the corresponding intervertebral disc. We also measured the approximate position and angle for the placement of the working cannula to reduce the risk of nerve root injury during Endo‐TLIF procedures in the Chinese population.

**Methods:**

This retrospective study was conducted at our institution between December 2021 and December 2022. A total of 115 patients suffering from low back pain were recruited for the study. For each participant, three‐dimensional (3D) vertebral models of the lumbar segments from L3 to S1 were constructed based on their CT images. The nerve root–disc distance, cannula insertion bypass distance and angle, foraminal height and width, exiting nerve root height, and nerve root–pedicle distance were measured. A paired t‐test was used to compare measurements between the left and right sides, while inter‐ and intraobserver reproducibility was assessed using the intraclass correlation coefficient (ICC).

**Results:**

From L3/4 to L5/S1 segments, the ideal cannula insertion distance range was 37.51 ± 4.91–120.38 ± 37.71 mm at L3/4; 42.38 ± 5.29–116.25 ± 27.22 mm at L4/5; and 37.78 ± 4.86–69.26 ± 12.64 mm at L5/S1. The appropriate cannula insertion angle range was 30.86° ± 5.05°–62.59° ± 6.66° at L3/4; 34.30° ± 4.73°–60.88° ± 7.34° at L4/5; and 35.89° ± 4.18°–47.65° ± 7.38° at L5/S1. The height of the intervertebral foramen (IVF) gradually decreased, and the width steadily increased. The exiting nerve root height and the nerve root–pedicle distance slightly decreased caudally.

**Conclusion:**

From L3/4 to L5/S1, the range of working cannula insertion distance and angle gradually decreased, and the exiting nerve root height occupying the IVF gradually increased. Our measurement can reduce the risk of nerve root injury caused by inserting the working cannula during Endo‐TLIF.

## Graphical abstract

This study introduces a novel approach for determining the approximate position and angle for placement of the working cannula. Observing a reduction in the insertion distance and angle from L3/4 to L5/S1 aims to reduce the risk of nerve root injury during Endo‐TLIF procedures.

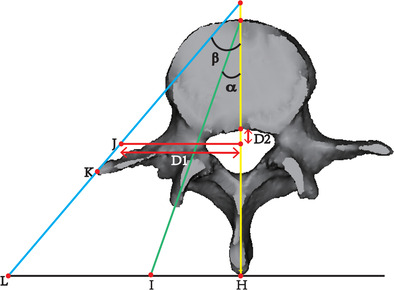

## Introduction

Lumbar degenerative diseases are a significant cause of surgery in adults. Open surgery is the most suitable option for significantly alleviating symptoms in patients with lumbar spinal disorders.^1^ However, open spinal fusions may lead to relatively extensive spine dissections, destabilized facet joints, prolonged hospital stays, and protracted recovery periods. Open transforaminal lumbar interbody fusion (TLIF) is a standard procedure, but up to 25.4% of patients experience surgical‐related complications.^2^ To overcome these limitations, minimally invasive TLIF was developed with the potential of further reducing the iatrogenic damage associated with the technique. However, this technique has the disadvantage of insufficient visualization, which may lead to incomplete nerve decompression or unstable lumbar fusion.^3^, ^4^, ^5^

Kambin and Gellman introduced percutaneous endoscopic lumbar discectomy (PELD) in 1983.^6^ Based on PELD, endoscopic‐assisted TLIF (Endo‐TLIF) has gradually been developed. Endo‐TLIF achieves nerve root decompression, intervertebral cage implantation, and fusion through the typical Kambin's triangle under endoscopic visualization.^7^, ^8^ While this technique provides effective transforaminal decompression with minimal tissue trauma and low rates of perioperative complications, it does have certain limitations. These include the potential for exiting nerve root extrusion and the challenges associated with endoscopic cage implantation.^9^, ^10^, ^11^ Due to bony constraints, a limited operating space, and a lack of cadaveric training opportunities, surgeons must rely heavily on clinical experience, which presents a steep learning curve.^12^ During the procedure, factors such as blind cannula placement, inadequate arthroplasty, or a restricted field of view can significantly increase the risk of nerve root injury.^13^ Only a few studies have offered theoretical anatomical guidance for Endo‐TLIF to date. To minimize these risks, surgeons must thoroughly understand the anatomical structures surrounding the intervertebral foramen (IVF). This study proposes a novel measurement to determine the approximate position and angle of the working cannula placement, providing surgeons with relevant anatomical data to reduce the risk of nerve root injury during Endo‐TLIF in the Chinese population.

The objectives of this study are twofold: (i) to construct three‐dimensional (3D) models of the lumbar vertebrae from L3 to S1 and determine the optimal position and angle for the working cannula, and (ii) to guide Endo‐TLIF surgeries by offering anatomical references for designing the working cannula.

## Methods

### Patient Selection

This retrospective study was approved by the Ethics Committee of the First Affiliated Hospital, College of Medicine, Zhejiang University, Hangzhou, China (No. 2023‐0255), and individual consent was waived due to the retrospective nature of this study. The patients were recruited between December 2021 and December 2022 at the institution. The inclusion criteria for participants were as follows: individuals aged 20–50 years, who were experiencing low back pain, had undergone 3–6 months of conservative treatment without practical results and had completed a lumbar spine computed tomography (CT) scan. The exclusion criteria were previous spinal surgery, disc prolapse, scoliosis, collapsed intervertebral space, spinal inflammation, tuberculosis, trauma, or gross deformity. A total of 115 patients (age range, 20–50 years; mean age, 40.4 years; 65 males and 50 females) were included.

### Clinical Measurements

CT images (GE Revolution EVO, USA) were acquired in the standard supine position (120 kV, automA, 0.7‐s duration, 32‐cm field of view, 512 × 512 matrix). The scan was acquired in the spiral scan mode, with the scan baseline parallel to the vertebral body. The scanning range was from L3 to S1, and the scanning layer thickness was 0.625 mm, with a spacing of 0.625 mm, a tube current of GE AutomA technology, and a tube voltage of 120 kV. A professional imaging workstation (TJAW47, GE, USA) performed the image postprocessing, and related measurements. CT images of the spinal segments were imported into the solid modeling software Mimics19 (Materialize, Leuven, Belgium) and Rhino7 (Robert McNeel & Associates, Seattle, WA, USA) to build the 3D anatomical vertebral body models of L3–S1 and measure the anatomical parameters of the lumbar IVF.

#### Transverse Plane

**FIGURE 1:**
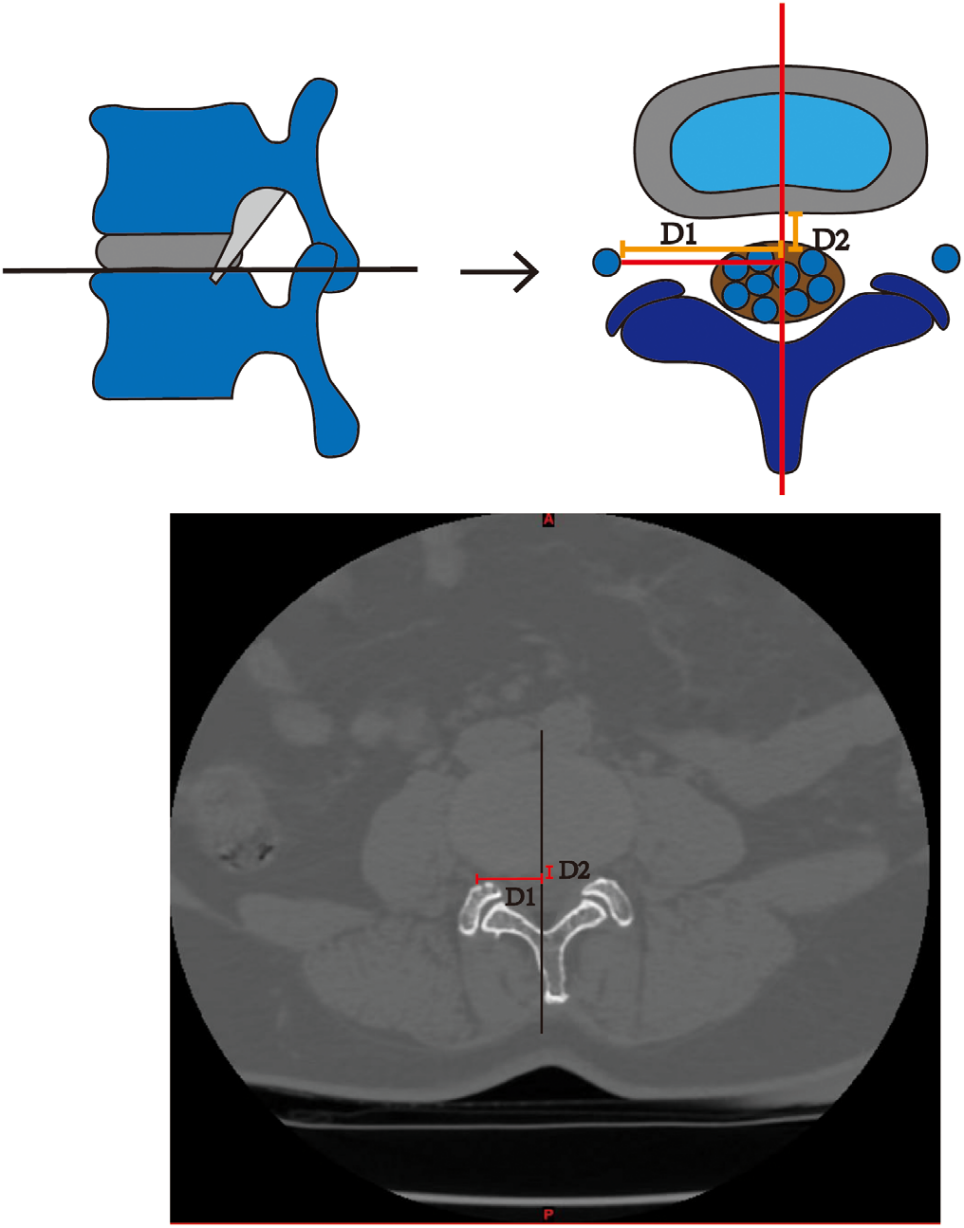
Example of the parameters D1 and D2 on the transverse plane of the computed tomography (CT) at the inferior margins of the target intervertebral disc. D1: Nerve root–disc horizontal distance; D2: Nerve root–disc vertical distance.

**FIGURE 2:**
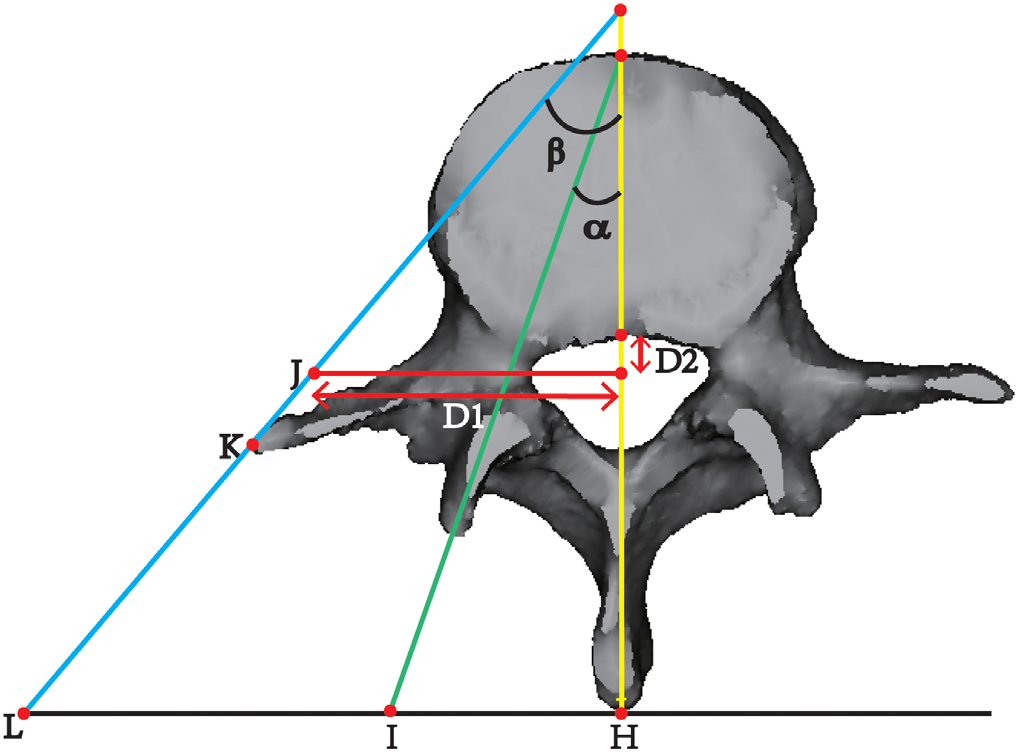
Example of the parameters calculated in the transverse plane on three‐dimensional (3D) lumbar vertebrae models. J: the medial edge point of the exiting nerve root (determined according to the D1 and D2); K: the vertex of the transverse process; The medial line: the tangent line of the superior articular process (green line); The outer line: the connecting line between J and K (blue line); The midline line: midline of the vertebral body (yellow line); The tangent line of spinous process (black line); L, I, and H: the outer line, the medial line and the middle line intersect with the tangent line of spinous process respectively at L, I, and H; HI (the cannula insertion minimum bypass distance): distance was formed by the intersection of the plane where the medial line is located and the plane where the vertebral midline is located in the spinous process tangent plane; HL (the cannula insertion maximum bypass distance): distance was formed by the intersection of the plane where the outer line is located and the plane where the vertebral midline is located in the spinous process tangent plane; *α* (the cannula insertion minimum angle): angle was formed between the plane of the medial line and the plane of the midline of the vertebral body; *β* (the cannula insertion maximum angle): angle was formed between the plane of outer line and the plane of midline of the vertebral body.

1. Nerve root–disc horizontal distance (D1): horizontal distance between the central inner margin of the exiting nerve root and the midline of the disc.
2. Nerve root–disc vertical distance (D2): vertical distance between the central inner margin of the exiting nerve root and the midline of the disc.
  The nerve root–disc vertical distance (D2) was measured below as positive readings (+mm) and above the disc as negative readings (−mm) (Figure 1).
3. The tangent line of the superior articular process was defined as the medial line because the dural sac was squeezed inward. The medial edge point of the exiting nerve root (J) was determined based on the nerve root–disc horizontal distance (D1) and nerve root–disc vertical distance (D2) measured on CT. The vertex of the transverse process (K) is the ideal position for observing the dural sac and nerve root from an oblique lateral angle and is also the perfect central position for the fusion cage to be placed from the oblique lateral side (excluding the transverse process variation). The connecting line of J and K is the outer line because the exiting nerve root is damaged further outward. The following measurements were obtained.
  - Cannula insertion minimum bypass distance (HI): distance was formed by the intersection of the plane where the medial line is located and the plane where the vertebral midline is situated in the spinous process tangent plane.
  - Cannula insertion maximum bypass distance (HL): distance was formed by the intersection of the plane where the outer line is located and the plane where the vertebral midline is situated in the spinous process tangent plane.
  - Cannula insertion minimum angle (*α*): angle was formed between the plane of the medial line and the plane of the midline of the vertebral body.
  - Cannula insertion maximum angle (*β*): angle was formed between the plane of the outer line and the plane of the midline of the vertebral body (Figure 2).

#### Pedicle Cutting Plane (Figure 3)

**FIGURE 3:**
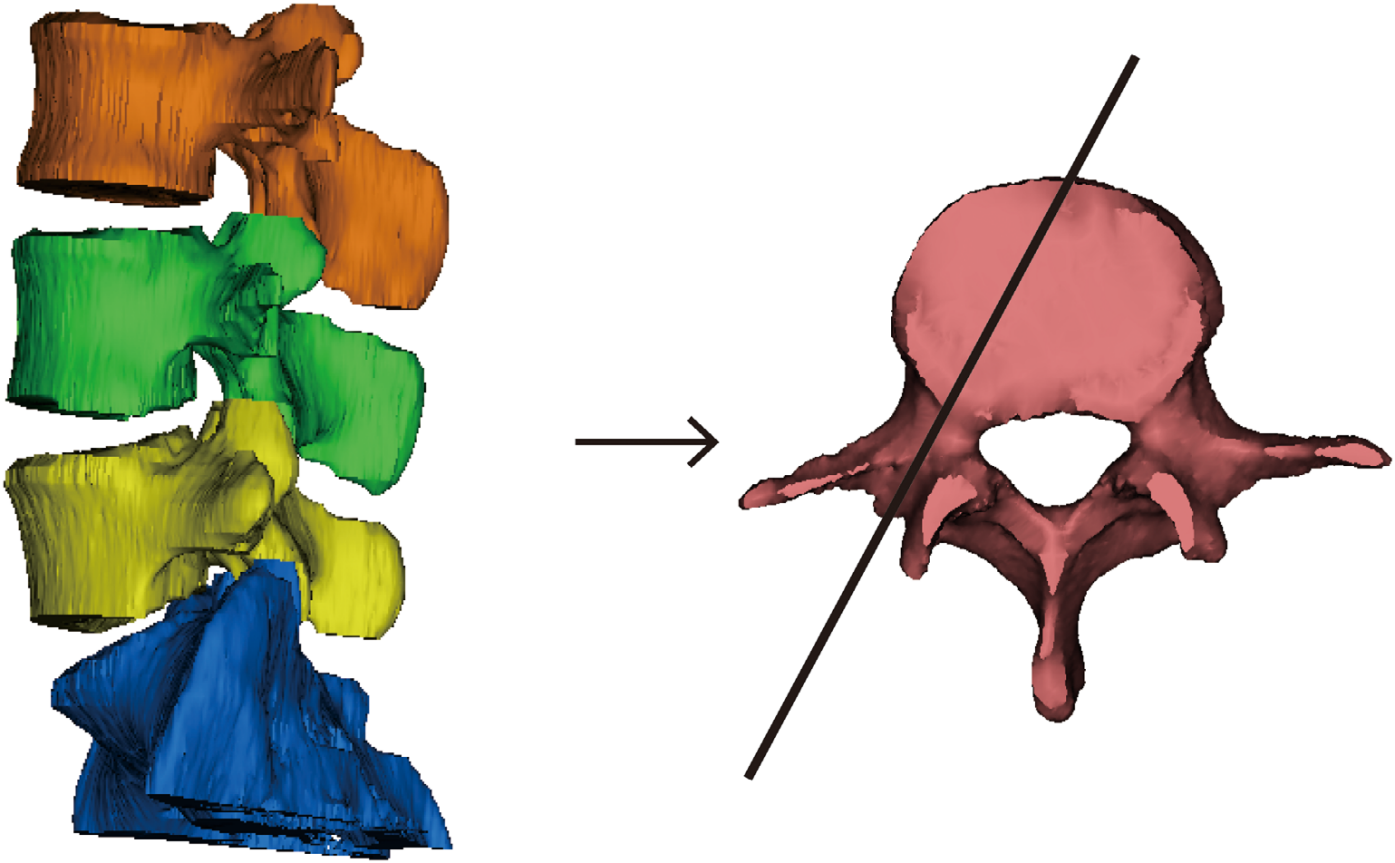
(Left) 3D lumbar vertebrae models of L3–S1; (Right) the pedicle cutting plane (black line; the vertical plane of the long axis of the pedicle).

**FIGURE 4:**
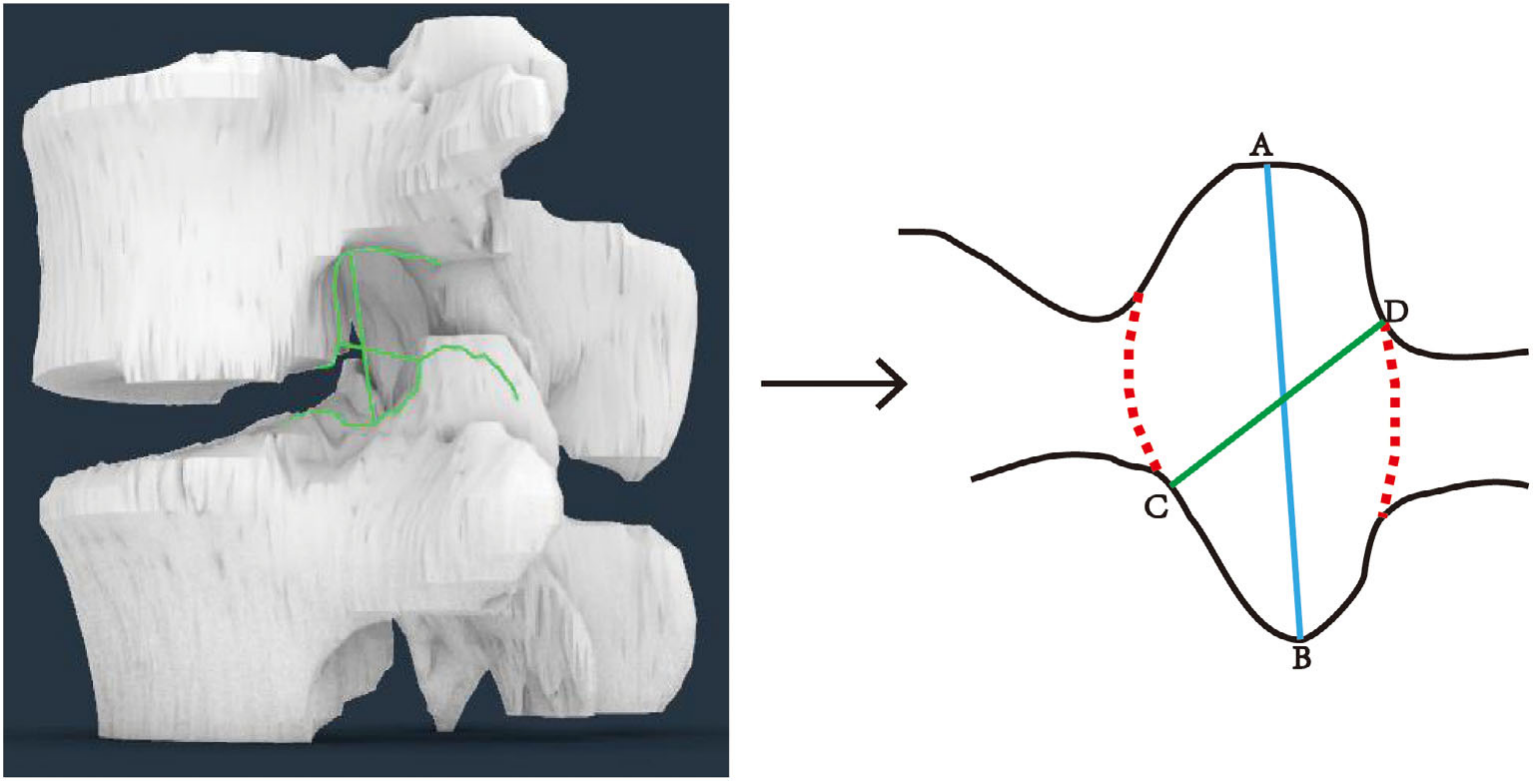
Examples of the intervertebral foramen (IVF) parameters measured on the pedicle cutting plane on three‐dimensional (3D) lumbar vertebrae models. The outline of IVF was formed according to the intersection line by the pedicle‐cutting plane. AB: Foraminal height (blue line); CD: Foraminal width (green line).

1. Foraminal height (AB): longest distance between the craniocaudal boundary (blue line).
2. Foraminal width (CD): shortest distance between the posterior–inferior and anterior–inferior corners of the cranial vertebrae (green line) (Figure 4).

#### Coronal Plane

**FIGURE 5:**
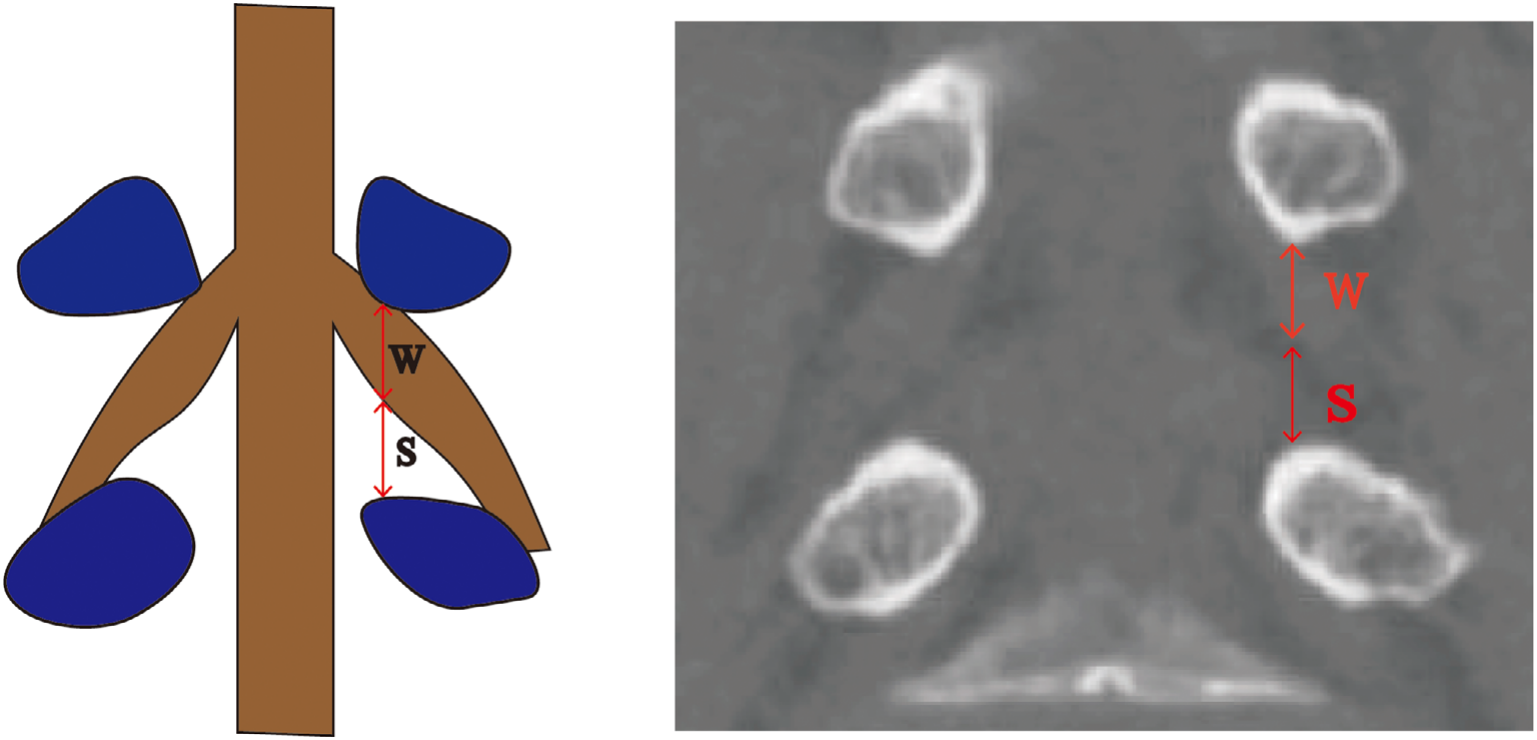
Examples of the parameters measured on the coronal plane of the computed tomography (CT) at the cutting plane of the pedicle. W: Exiting nerve root height; S: Nerve root–pedicle distance.

1. Exiting nerve root height (W): distance between the inferior margin of the pedicle and the inferior margin of the exiting nerve root.
2. Nerve root–pedicle distance (S): distance between the inferior margin of the exiting nerve root and the superior margin of the inferior pedicle (foraminal height minus the exiting nerve root height) (Figure 5).

## Statistical Methods

All data obtained in this study were collected by two senior doctors in the Department of Radiology at the First Affiliated Hospital of Zhejiang University. Statistical analyses were performed using SPSS software (version 26.0; IBM, USA). Mean values and standard deviations were calculated. A paired t‐test was used to compare measurements between the left and right sides. Inter‐ and intraobserver reproducibility were calculated using the intraclass correlation coefficient (ICC). Statistical significance was set at *p* < 0.05.

## Results

The average values for the respective planes and levels are summarized in Tables 1, 2, 3. Both planes had good intragroup consistency in the measured parameters, with ICC values ranging from 0.816 to 0.958.

**TABLE 1:** Transverse plane measurements

Level/Side	Nerve root–disc horizontal distance (mm)	Nerve root–disc vertical distance (mm)	Cannula insertion maximum bypass distance (mm)	Cannula insertion minimum bypass distance (mm)	Cannula insertion maximum angle (°)	Cannula insertion minimum angle (°)
L3–L4						
Bilateral	20.13 ± 2.07	0.93 ± 1.98	120.38 ± 37.71	37.51 ± 4.91	62.59 ± 6.66	30.86 ± 5.05
Left	20.61 ± 2.28	0.92 ± 2.13	116.96 ± 35.96	37.10 ± 5.41	61.99 ± 7.45	30.64 ± 5.69
Right	19.66 ± 2.25*	0.94 ± 2.08	123.81 ± 46.88	37.93 ± 5.21	63.19 ± 7.44	31.09 ± 5.13
L4–L5						
Bilateral	23.80 ± 2.05	−4.85 ± 2.64	116.25 ± 27.22	42.38 ± 5.29	60.88 ± 7.34	34.30 ± 4.73
Left	24.27 ± 2.31	−4.92 ± 2.94	112.63 ± 26.90	41.91 ± 5.93	59.91 ± 7.77	33.71 ± 5.32
Right	23.33 ± 2.38*	−4.78 ± 2.79	119.86 ± 32.14	42.86 ± 5.49	61.84 ± 8.06*	34.90 ± 4.88*
L5–S1						
Bilateral	22.91 ± 2.28	−9.61 ± 2.69	69.26 ± 12.64	37.78 ± 4.86	47.65 ± 7.38	35.89 ± 4.18
Left	23.48 ± 2.54	−9.50 ± 2.73	68.66 ± 13.94	37.87 ± 4.90	49.99 ± 8.24	35.67 ± 4.53
Right	22.34 ± 2.56*	−9.71 ± 2.87	69.86 ± 13.38	37.70 ± 5.81	48.31 ± 7.98*	36.12 ± 4.58

*Note*: Values are mean ± SD. The paired *t*‐test shows that compared with the left side; *p < 0.05.

**Table 2:** Pedicle‐cutting plane measurements

Level/Side	Foraminal height (mm)	Foraminal width (mm)
L3–L4		
Bilateral	18.87 ± 1.83	8.32 ± 1.68
Left	18.81 ± 2.08	8.40 ± 1.74
Right	18.93 ± 1.89	8.23 ± 1.85
L4–L5		
Bilateral	17.96 ± 1.81	8.41 ± 1.87
Left	18.08 ± 2.07	8.56 ± 2.10
Right	17.84 ± 1.91	8.27 ± 1.97
L5–S1		
Bilateral	15.92 ± 1.84	11.68 ± 2.18
Left	15.86 ± 1.99	11.86 ± 2.28
Right	15.97 ± 1.98	11.49 ± 2.25

*Note*: Values are mean ± SD.

**TABLE 3:** Coronal plane measurements

Level/Side	Exiting nerve root height (mm)	Nerve root–pedicle distance (mm)
L3–L4		
Bilateral	7.81 ± 1.31	11.02 ± 1.80
Left	7.87 ± 1.47	10.90 ± 2.10
Right	7.74 ± 1.55	11.15 ± 1.98
L4–L5		
Bilateral	7.35 ± 1.24	10.61 ± 1.77
Left	7.61 ± 1.43	10.47 ± 2.11
Right	7.08 ± 1.37*	10.75 ± 1.87
L5–S1		
Bilateral	7.69 ± 1.17	8.23 ± 1.69
Left	7.82 ± 1.21	8.04 ± 1.88
Right	7.56 ± 1.40	8.41 ± 1.89

*Note*: Values are mean ± SD. The paired *t*‐test shows that compared with the left side; *p < 0.05.

### Transverse Plane Measurements

The mean value of the nerve root–disc horizontal distance was 20.13 ± 2.07 mm at L3/4, 23.80 ± 2.05 mm at L4/5, and 22.91 ± 2.28 mm at L5/S1. From L3/4 to L5/S1, notable differences were observed between the right and left sides of the nerve root–disc horizontal distance. The mean value of the nerve root–disc vertical distance was 0.93 ± 1.98 mm at L3/4, −4.85 ± 2.64 mm at L4/5, and − 9.61 ± 2.69 mm at L5/S1. The average value of cannula insertion maximum bypass distance (HL) was 120.38 ± 37.71 mm at L3/4, 116.25 ± 27.22 mm at L4/5, and 69.26 ± 12.64 mm at L5/S1. The average value of cannula insertion minimum bypass distance (HI) was 37.51 ± 4.91 mm at L3/4, 42.38 ± 5.29 mm at L4/5, and 37.78 ± 4.86 mm at L5/S1. The mean cannula insertion minimum angle (α) at L3/4 was 30.86° ± 5.05°, at L4/5 was 34.30° ± 4.73°, and at L5/S1 was 35.89° ± 4.18°. The mean cannula insertion maximum angle (*β*) at L3/4 was 62.59° ± 6.66°, at L4/5 was 60.88° ± 7.34°, and at L5/S1 was 47.65° ± 7.38°. For the left and right sides of L4/5 and L5/S1, the cannula insertion angle (*α*/*β*) result was statistically significant, and the difference values were 1.93° (*β* at L4/5),1.68° (*β* at L5/S1), and 1.19° (*α* at L4/5) respectively. As shown in Table 1, there were no significant differences between the left and right sides of each group with respect to the nerve root–disc vertical distance, cannula insertion maximum bypass distance, and cannula insertion minimum bypass distance.

### Pedicle Cutting Plane Measurements

The mean value of foraminal height was 18.87 ± 1.83 mm at L3/4, 17.96 ± 1.81 mm at L4/5, and 15.92 ± 1.84 mm at L5/S1. The mean value of foraminal width was 8.32 ± 1.68 mm at L3/4, 8.41 ± 1.87 mm at L4/5, and 11.68 ± 2.18 mm at L5/S1. As shown in Table 2, there were no significant differences in the height or width of the foramina between the left and right sides of each segment.

### Coronal Plane Measurements

The average values of the exiting nerve root height were 7.81 ± 1.31 mm at L3/4, 7.35 ± 1.24 mm at L4/5, and 7.69 ± 1.17 mm at L5/S1. Only the results for the left and right sides of the L4/5 level were significantly different, with a difference of 0.53 mm. The average value of nerve root–pedicle distance was 11.02 ± 1.80 mm at L3/4, 10.61 ± 1.77 mm at L4/5, and 8.23 ± 1.69 mm at L5/S1. As shown in Table 3, no significant differences were found when comparing each group's left and right measurements.

## Discussion

### Main Findings

This study used CT data from 115 patients with low back pain to construct 3D lumber spine models, specifically from L3 to S1, and conducted detailed anatomical measurements. Generally, as the spinal segment progressed downwards, the safe insertion range and angle for the working cannula decreased. Concurrently, the height of the IVF decreased while its width increased. Additionally, both the height of the exiting nerve root and the nerve root–pedicle distance showed a slight decrease in the lower segments.

### Working Cannula Placement Selection

The primary working area during Endo‐TLIF procedures is the IVF space. Understanding the position of the exiting nerve root relative to the inferior margin of the disc can help reduce the risk of nerve root injury. We measured the horizontal and vertical distances from the exiting nerve root to the midline of the intervertebral disc in the transverse section of the inferior margin of the disc to determine the location of the exiting nerve root. Our results showed that the nerve root–disc horizontal and vertical distances gradually increased caudally. Notably, from L3/4 to L5/S1, there was a significant difference between the left and right sides in the horizontal distance from the nerve root to the intervertebral disc, both being larger on the left side. This is a common issue in single‐center retrospective studies and requires verification through an expanded sample size. Typically, surgeons assess the position of the working cannula using multiple intraoperative fluoroscopies; however, this increases radiation exposure. Hirayama *et al*. determined the insertion angle of the working cannula by rotating a 3D CT/MR fusion image to match the angle of the largest Kambin triangle working area.^14^ Currently, no anatomical data related to the position and angulation of the working cannula have been reported in the literature. Our team proposed a novel measurement to determine the approximate position and angle of the working cannula, as illustrated in Figures 1 and 2. The ideal safe insertion distance range was 37.51 ± 4.91–120.38 ± 37.71 mm at L3/4, 42.38 ± 5.29–116.25 ± 27.22 mm at L4/5, and 37.78 ± 4.86–69.26 ± 12.64 mm at L5/S1. The operation space at L5/S1 is limited, and the field of view is insufficient. Moreover, caution is needed for patients with L5–S1 intervertebral disc herniation and a high iliac crest. The obstruction caused by the high iliac crest can hinder the entry of the working cannula into the target area and may even result in puncture failure. Surgeons should endeavor to remove the superior articular process as much as possible to minimize the risk of nerve root damage. Additionally, the insertion angle of the working cannula is 30.86 ± 5.05–62.59 ± 6.66° at L3/4, 34.30 ± 4.73–60.88 ± 7.34° at L4/5, and 35.89 ± 4.18–47.65 ± 7.38° at L5/S1.

### IVF Size Change Trend

The height and width of the IVF in our study were measured at the pedicle cutting plane based on 3D CT reconstruction, which has the narrowest cross‐sectional area of the IVF and is most consistent with the surgical approach.^15^ Our results showed that foraminal height decreased caudally, while it was the opposite for width. Torun et al.^16^ reported that the mean values of the foraminal height and width were 19.4 and 8.9 mm, respectively, in a study of 15 cadavers. In contrast to our findings, this group measured the maximum foraminal height at the L5/S1. Moreover, Evins et al.^17^ reported that the average foraminal height on CT scans of cadavers was 17.5 mm, and the trend decreased with the downward segment, which is consistent with our study. This cadaver study has some limitations. To fully expose the field of vision, the tissues around the anatomical structures suffered additional damage, especially at the L5/S1 level.^18^ In addition, formalin immersion in cadavers affects the anatomical relationship by reducing the internal tension of tissues.^19^ In a study by Hurday et al.,^20^ the foraminal height measured using magnetic resonance imaging (MRI) was 21.55 mm, gradually decreasing from L2/3 to L5/S1. Radiological measurements can avoid structural damage and better reflect the authenticity of the data. Hurday's team measured in the sagittal plane, while we measured in the pedicle cutting plane, which can better provide anatomical information that can be used intraoperation. Without considering the exiting nerve root, IVF width was the primary limiting factor affecting the placement of the working cannula.

### The Course of Nerve Roots

A coronal plane study showed that from L3 to S1, the route of the nerve root in the IVF gradually becomes vertical.^19^ The exiting nerve root of the IVF area passes the disc between the middle and lateral boundaries of the pedicle, limiting the safe insertion of the working cannula here.^20^ Therefore, it is essential to understand the course and distribution of exiting nerve roots in IVF. We measured the height of the exiting nerve root at the pedicle cutting plane, enabling complete observation of the nerve root. The height of the exiting nerve root slightly decreased caudally and could be arranged from largest to smallest as follows: L3/4 (7.81 ± 1.31 mm) > L5/S1 (7.69 ± 1.17 mm) > L4/5 (7.35 ± 1.24 mm). According to the previously obtained height of the IVF, the exiting nerve root height thus occupied more than 1/2 of the IVF size at L5/S1. Notably, there was a prominent difference in the height of the exiting nerve root between the left and right sides at L4/5. Occupation in the foramen measures nerve root compression; higher nerve root occupation indicates higher nerve root compression.^21^ Hurday et al.^20^ used MRI to demonstrate that the nerve root–pedicle distance gradually increased with the segment caudally, which contrasts with our results. This discrepancy may be attributed to the utilization of different reference planes. We selected the pedicle‐cutting plane, where a certain angle was observed between the pedicle‐cutting plane and the sagittal plane, thus resulting in variations in the reflected anatomical data. Additionally, the distance from the exiting nerve root to the pedicle, as measured by Zhang et al. in the rotating coronal plane, gradually decreased caudally.^1^

### Limitations

Several limitations of this study should be considered. First, uncertainty in the density of nerve roots and surrounding tissue on CT scans may result in measurement errors. Second, the age range of the study population was limited, and a larger sample size is needed for better generalization. Finally, due to the technical constraints of this study, nerve root reconstruction was not possible, and some errors may have occurred if the imaging data had been applied to the 3D model.

### Prospects of Clinical Application

In Endo‐TLIF surgery, the placement of the working cannula is crucial for the procedure's success. The imaging parameters outlined in this study play a significant role in developing surgical plans and guiding intraoperative maneuvers. These parameters not only aid surgeons in accurately positioning the working cannula to ensure it reaches the target surgical area without causing damage to surrounding tissues, but they also enhance the overall safety and efficiency of the surgery. Additionally, they contribute to better exposure to the surgical field, facilitating nerve decompression, disc resection, endplate preparation, and the placement of fusion devices. However, some challenges remain. Variations in the lumbar anatomical structures of different patients can impact the accuracy of the working cannula's placement. Furthermore, the surgeon's experience and technical proficiency directly influence the precision and stability of cannula placement. As technology advances and clinical experience accumulates, the prospects for Endo‐TLIF surgery in clinical practice are expected to become even more promising.

## Conclusion

From L3/4 to L5/S1, the range of working cannula insertion distance and angle gradually decreased, and the exiting nerve root height occupying the IVF gradually increased. Our measurement can reduce the risk of nerve root injury caused by inserting the working cannula during Endo‐TLIF.

## Author Contributions

Concept/idea/research design, analysis, and interpretation of data: Conghui Zhou, Mengran Jin, and Junsong Wu. Acquisition of data: Conghui Zhou, Junsheng Lou, Yunpeng Fan, and Honghao Shen. Writing/review/editing of manuscript: Conghui Zhou, Junsheng Lou, Mengran Jin. Acquisition of funding and providing facilities/equipment: Junsong Wu, Mengran Jin, and Ziyi Guo. Providing facilities/equipment: Junsong Wu, Mengran Jin, and Ziyi Guo. Providing subjects: Mengran Jin and Junsong Wu. Final approval of the manuscript: All authors.

## Conflict of Interest

The author(s) declared no potential conflicts of interest with respect to the research, authorship, and/or publication of this article.

## Ethics Statement

Due to the retrospective nature of the study, informed consent was waived. This study was approved by the Ethics Committee of the First Affiliated Hospital, College of Medicine, Zhejiang University, Hangzhou, China (No. 2023‐0255).

## Funding Information

This project was supported by the Zhejiang Provincial Natural Science Foundation of China (Grant number: LY22H150002 to Junsong Wu), Zhejiang Provincial Natural Science Foundation of China (Grant number: LHDMY23H100002 to Mengran Jin), National Clinical Research Center for Orthopedics, Sports Medicine &Rehabilitation of China (Grant number: 2021‐NCRC‐CXJJ‐PY‐41 to ZiYi Guo).

## Data Availability

The raw data supporting the conclusions of this article will be made available by the authors, without undue reservation.

## References

[1] Zhang KH , Zhang WH , Xu BS , Dong XM , Guo L , du LL , et al. CT‐based morphometric analysis of approach of percutaneous transforaminal endoscopic lumbar interbody fusion. Orthop Surg. 2019;11(2):212–220. 30895721 10.1111/os.12434 PMC6594482

[2] Tormenti MJ , Maserati MB , Bonfield CM , Gerszten PC , Moossy JJ , Kanter AS , et al. Perioperative surgical complications of transforaminal lumbar interbody fusion: a single‐center experience. J Neurosurg Spine. 2012;16(1):44–50. 21999389 10.3171/2011.9.SPINE11373

[3] Foley KT , Holly LT , Schwender JD . Minimally invasive lumbar fusion. Spine (Phila Pa 1976). 2003;28(15 Suppl):S26–S35. 12897471 10.1097/01.BRS.0000076895.52418.5E

[4] Guan J , Bisson EF , Dailey AT , Hood RS , Schmidt MH . Comparison of clinical outcomes in the National Neurosurgery Quality and outcomes database for open versus minimally invasive transforaminal lumbar interbody fusion. Spine (Phila Pa 1976). 2016;41(7):E416–E421. 26536435 10.1097/BRS.0000000000001259

[5] Heemskerk JL , Oluwadara Akinduro O , Clifton W , Quinones‐Hinojosa A , Abode‐Iyamah KO . Long‐term clinical outcome of minimally invasive versus open single‐level transforaminal lumbar interbody fusion for degenerative lumbar diseases: a meta‐analysis. Spine J. 2021;21(12):2049–2065. 34273567 10.1016/j.spinee.2021.07.006

[6] Jasper GP , Francisco GM , Telfeian AE . Endoscopic transforaminal discectomy for an extruded lumbar disc herniation. Pain Physician. 2013;16(1):E31–E35. 23340542

[7] Lv Y , Chen M , Wang SL , Qin RJ , Ma C , Ding QR , et al. Endo‐TLIF versus MIS‐TLIF in 1‐segment lumbar spondylolisthesis: a prospective randomized pilot study. Clin Neurol Neurosurg. 2022;212:107082. 34902752 10.1016/j.clineuro.2021.107082

[8] Zhao XB , Ma HJ , Geng B , Zhou HG , Xia YY . Early clinical evaluation of percutaneous full‐endoscopic transforaminal lumbar interbody fusion with pedicle screw insertion for treating degenerative lumbar spinal stenosis. Orthop Surg. 2021;13(1):328–337. 33426744 10.1111/os.12900 PMC7862160

[9] Wu W , Yang S , Diao W , Wang D , Guo Y , Yan M , et al. Analysis of clinical efficacy of endo‐LIF in the treatment of single‐segment lumbar degenerative diseases. J Clin Neurosci. 2020;71:51–57. 31843435 10.1016/j.jocn.2019.11.004

[10] Shi L , Ding T , Shi Y , Wang F , Wu C . Comparison of the outcomes of minimally invasive transforaminal lumbar interbody fusion and endoscopic transforaminal lumbar interbody fusion for lumbar degenerative diseases: a retrospective matched case‐control study. World Neurosurg. 2022;167:e1231–e1240. 36096389 10.1016/j.wneu.2022.09.013

[11] Ge M , Zhang Y , Ying H , Feng C , Li Y , Tian J , et al. Comparison of hidden blood loss and clinical efficacy of percutaneous endoscopic transforaminal lumbar interbody fusion and minimally invasive transforaminal lumbar interbody fusion. Int Orthop. 2022;46(9):2063–2070. 35723702 10.1007/s00264-022-05485-z PMC9372117

[12] Ahn Y , Youn MS , Heo DH . Endoscopic transforaminal lumbar interbody fusion: a comprehensive review. Expert Rev Med Devices. 2019;16(5):373–380. 31044627 10.1080/17434440.2019.1610388

[13] Lee SH , Kang HS , Choi G , Kong BJ , Ahn Y , Kim JS , et al. Foraminoplastic ventral epidural approach for removal of extruded herniated fragment at the L5‐S1 level. Neurol Med Chir (Tokyo). 2010;50(12):1074–1078. 21206181 10.2176/nmc.50.1074

[14] Hirayama J , Hashimoto M , Sakamoto T . Clinical outcomes based on preoperative Kambin's triangular working zone measurements on 3D CT/MR fusion imaging to determine optimal approaches to transforaminal endoscopic lumbar Diskectomy. J Neurol Surg A Cent Eur Neurosurg. 2020;81(4):302–309. 31962355 10.1055/s-0039-3400752

[15] Fujiwara A , An HS , Lim TH , Haughton VM . Morphologic changes in the lumbar intervertebral foramen due to flexion‐extension, lateral bending, and axial rotation: an in vitro anatomic and biomechanical study. Spine (Phila Pa 1976). 2001;26(8):876–882. 11317109 10.1097/00007632-200104150-00010

[16] Torun F , Dolgun H , Tuna H , Attar A , Uz A , Erdem A . Morphometric analysis of the roots and neural foramina of the lumbar vertebrae. Surg Neurol. 2006;66(2):148–151. discussion 151. 16876606 10.1016/j.surneu.2006.02.041

[17] Evins AI , Banu MA , Njoku I Jr , Elowitz EH , Härtl R , Bernado A , et al. Endoscopic lumbar foraminotomy. J Clin Neurosci. 2015;22(4):730–734. 25744073 10.1016/j.jocn.2014.10.025

[18] Arslan M , Comert A , Acar HI , Ozdemir M , EIhan A , Tekdemir I , et al. Nerve root to lumbar disc relationships at the intervertebral foramen from a surgical viewpoint: An anatomical study. Clin Anat. 2012;25(2):218–223. 21671286 10.1002/ca.21213

[19] Arslan M , Comert A , Acar HI , Ozdemir M , EIhan A , Tekdemir I , et al. Neurovascular structures adjacent to the lumbar intervertebral discs: an anatomical study of their morphometry and relationships. J Neurosurg Spine. 2011;14(5):630–638. 21332275 10.3171/2010.11.SPINE09149

[20] Hurday Y , Xu B , Guo L , Cao Y , Wan Y , Jiang H , et al. Radiographic measurement for transforaminal percutaneous endoscopic approach (PELD). Eur Spine J. 2017;26(3):635–645. 26922736 10.1007/s00586-016-4454-z

[21] Modi HN , Suh SW , Song HR , Yang JH . Lumbar nerve root occupancy in the foramen in achondroplasia: a morphometric analysis. Clin Orthop Relat Res. 2008;466(4):907–913. 18259829 10.1007/s11999-008-0142-6 PMC2504658

